# Pre-anesthetic Ultrasonography of the Subclavian Vein and Inferior Vena Cava for Predicting Hypotension After General Anesthesia Induction in Patients Undergoing Abdominopelvic Surgeries: A Prospective Observational Study

**DOI:** 10.7759/cureus.105005

**Published:** 2026-03-10

**Authors:** Sarita Sah, Gegal Pruthi, Mayank Gupta, Akash Mishra, Soumya Swaroop Sahoo, Anju Grewal

**Affiliations:** 1 Anaesthesiology, All India Institute of Medical Sciences, Rishikesh, Rishikesh, IND; 2 Anaesthesiology, All India Institute of Medical Sciences, Bathinda, Bathinda, IND; 3 Biostatistics, Institute of Medical Sciences, Banaras Hindu University, Varanasi, IND; 4 Community and Family Medicine, All India Institute of Medical Sciences, Bathinda, Bathinda, IND

**Keywords:** anesthesia, hypotension, inferior vena cava, subclavian vein, ultrasound

## Abstract

Background: Post-induction hypotension (PIH) jeopardizes vital organ perfusion and is associated with perioperative morbidity and mortality. This study aimed to evaluate and compare the diagnostic accuracy of preoperative ultrasonography (USG) measurement of the inferior vena cava (IVC) and subclavian vein (SCV) for predicting PIH following induction of general anesthesia (GA) in patients undergoing abdominopelvic surgeries.

Methods: A prospective observational study was conducted on 57 patients of North Indian ethnicity undergoing elective abdominopelvic surgeries. IVC and SCV diameters and collapsibility index (CI) were measured using USG before induction of GA. The study focused on comparing these measurements with the incidence of PIH.

Results: The IVC-CI (p <0.001), SCV-CI (p <0.001), and the minimum diameter of the IVC (p = 0.008) were significant predictors of PIH. The optimal cut-off values for predicting PIH were 27.91% and 35.59% for IVC-CI and SCV-CI, respectively. SCV-CI exhibited diagnostic accuracy comparable to that of IVC-CI in predicting PIH, demonstrating higher specificity (97% vs. 90.3%) but marginally lower sensitivity (69.2% vs. 73.1%). The time taken to measure USG-guided SCV parameters was significantly less compared to that of IVC (111.58± 25.91 vs. 155.91 ± 35.77 seconds; p < 0.001). No significant correlations were found between PIH and the maximum IVC or maximum or minimum SCV diameters.

Conclusion: IVC-CI and SCV-CI showed comparable diagnostic accuracy in predicting PIH under a standardized induction protocol. Early identification of high-risk patients may enable anticipatory hemodynamic management. SCV visualization and assessment were faster than IVC, making SCV-CI a practical alternative when IVC imaging is challenging or when rapid evaluation is needed, although multicenter studies are needed for validation.

## Introduction

Post-induction hypotension (PIH) is a common complication after intravenous (IV) anesthetic agents owing to their cardio-depressant and sympatholytic activity [1]. Hypovolemia, pre-existing comorbidities, nil per os (NPO) hours, chronic use of antihypertensive drugs like angiotensin receptor blockers (ARBs) or angiotensin-converting enzyme inhibitors (ACEIs) are known to exacerbate PIH [2].

Hypotension reduces oxygen delivery and perfusion of vital organs, causing myocardial infarction, cerebrovascular accident, acute kidney injury, longer hospital stays, postoperative delirium and perioperative morbidity and mortality [1,3]. Therefore, a timely and accurate assessment of intravascular volume status is essential to minimize hemodynamic dysfunction [4]. Hypovolemia can be identified preoperatively by static & dynamic techniques. Static parameters like mean arterial pressure (MAP), central venous pressure, pulmonary artery occlusion pressure, and left ventricular end-diastolic area index do not provide real-time values and are invasive with increased risk of infection, thrombosis and iatrogenic complications [1,5], whereas dynamic parameters like pulse pressure variation, plethysmographic variability index, stroke volume variation, and inferior vena cava (IVC) or subclavian vein (SCV) diameter and collapsibility index (CI) offer real-time assessment of volume status and predict fluid responsiveness [1].

Ultrasonography (USG) assessment of the IVC does not directly predict arterial pressure but offers a non-invasive, reliable method for estimating volume status, helps identify patients at higher risk of developing PIH, and guides pre-anesthetic fluid management, thereby minimizing risks linked to invasive monitoring [1,4,6]. However, visualization of the IVC may be limited in up to 16% of patients [7,8], particularly in those with abdominal distension, masses, tenderness, morbid obesity, IVC obstruction (due to devices, thrombus, or filters), abdominal scars/incisions, open wounds and the measurements can also be altered by factors like cardiac tamponade, severe valvular stenosis, increased or decreased tidal volume, and term pregnancy [9-11]. Previous studies have found SCV-CI as a valuable adjunct or alternative to IVC-CI for evaluating intravascular volume status in patients with kidney or acute heart failure or admitted to the surgical intensive care unit (SICU) [9,10,12,13]. Evidence comparing IVC and SCV parameters for predicting PIH under general anesthesia (GA) remains limited [11,14,15].

To date, no study has directly compared the diagnostic accuracy of SCV and IVC diameter and CI in the North Indian population undergoing abdominopelvic surgery. Therefore, the present study was designed with the aim of evaluating pre-anesthetic USG-guided IVC and SCV parameters. The primary objective was to compare their diameter and CI for predicting PIH following the induction of GA in adult patients from the North Indian population undergoing abdominopelvic surgeries. The secondary objective was to calculate and compare the total time required to measure the USG parameters of IVC and SCV, thereby assessing their feasibility in a peri-induction setting. Early identification of patients at risk of PIH may allow anesthesiologists to implement preventive strategies such as intravascular volume optimization, careful titration of induction agents, preparation for early vasopressor use, and closer hemodynamic monitoring.

## Materials and methods

This prospective observational study was conducted from May 2023 to April 2024 after approval from the institutional ethics committee of All India Institute of Medical Sciences, Bathinda (IEC/AIIMS/BTI/268) and registration with the Clinical Trials Registry-India (CTRI/2023/04/051537; dated 12/4/2023). After taking written informed consent, patients aged 18-60 years belonging to the North Indian population, American Society of Anesthesiologists (ASA) Physical status I and II [16], scheduled for abdominopelvic surgeries under GA were included in the study. Abdominopelvic surgeries were defined as elective open or laparoscopic procedures involving organs within the abdominal or pelvic cavities, including gastrointestinal, hepatobiliary, gynecological, and urological operations. The exclusion criteria were patients allergic to any of the study drugs, moderate to severe hepatic, respiratory, renal or cardiac diseases, ascites or abdominal masses, pregnancy, body mass index (BMI) > 35 kg/m^2^, combined epidural and GA, preoperative hypotension (systolic blood pressure (SBP) < 70mmHg) or hypertensive crisis (blood pressure (BP) > 180/120 mmHg), already on mechanical ventilation and anticipated or unanticipated difficult airway.

Intervention technique

All patients were kept NPO for six hours prior to surgery [17]. Patients taking ACEIs or ARBs were asked to stop these drugs on the morning of surgery in accordance with the European Society of Anesthesiology and European Society of Cardiology guidelines [18]. On arrival to the pre-operative area, an 18-gauge IV cannula was secured and connected to Ringer’s lactate drip at a rate of 2 ml/kg/hour. 

**Figure 1:**
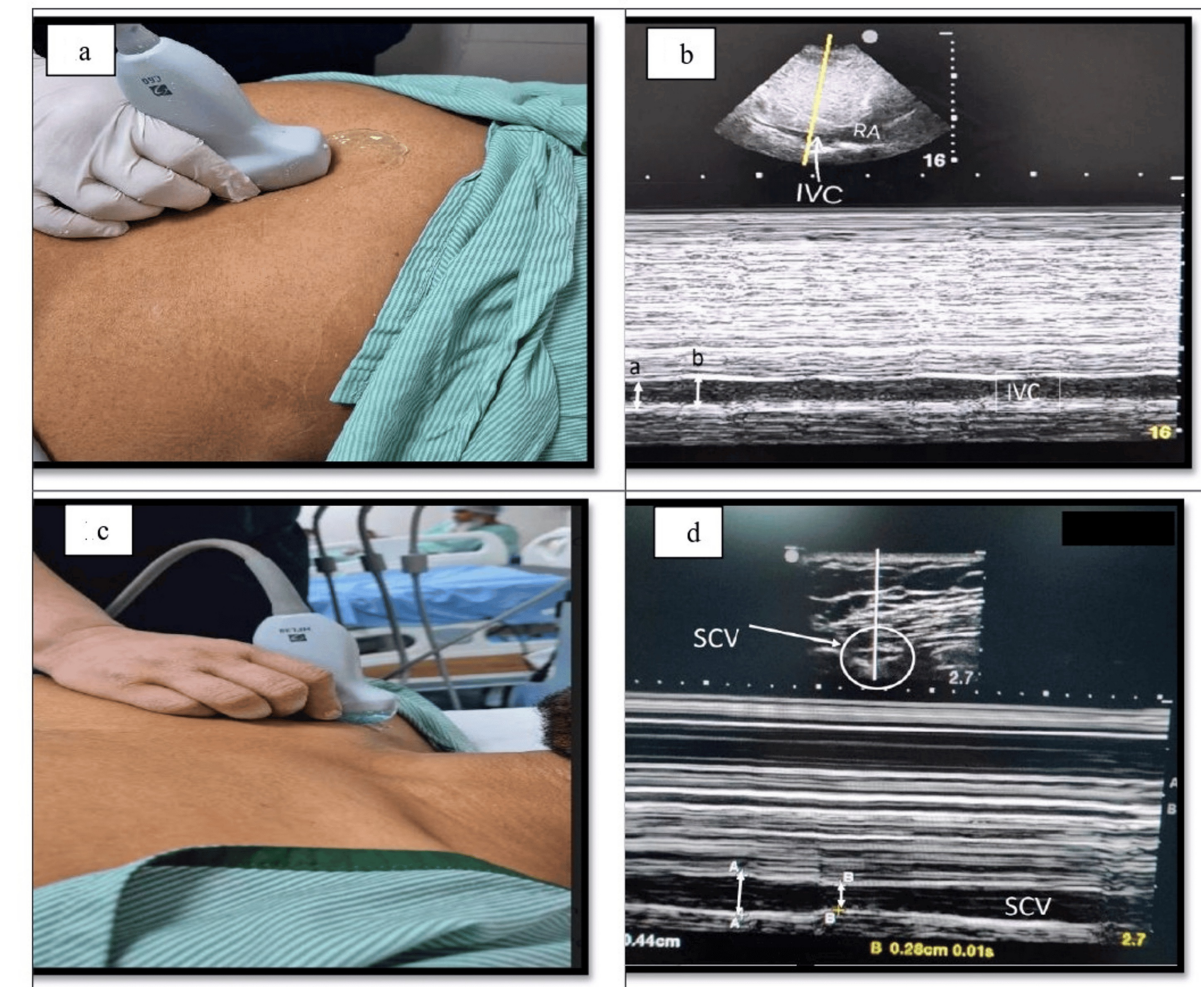
Ultrasound assessment of inferior vena cava (IVC) and subclavian vein (SCV) for dynamic diameter measurement a: Ultrasound (standard curvilinear) probe position in the subcostal region for IVC visualization. b: M-Mode tracing of the IVC, where the minimum (a) and maximum (b) diameters (marked by the double-sided white arrow) are measured perpendicular to the inner border of the vessels shown by the white line. c: Ultrasound (standard linear) probe position in the delto-pectoral groove for visualization of SCV. d: M Mode tracing of the SCV, where maximum (A) and minimum (B) diameters (marked by the double-sided white arrow) are measured perpendicular to the inner border of the vessels shown by the white line.

USG evaluation was done by a single anesthesiologist, experienced in perioperative ultrasonography, with prior training in vascular USG assessment. The measurements of IVC and SCV were performed using a low-frequency (2-5 MHz) curvilinear and high-frequency (6-13MHz) linear probes, respectively (Edge II, FUJIFILM Sonosite Inc., USA). All USG measurements were obtained prior to induction of anesthesia with the patient lying supine and breathing spontaneously. The IVC diameter measurements were taken in M-mode via subcostal approach at 2-3 cm distal to the right atrium using paramedian long-axis view (Figures 1a, 1b). For right SCV measurements, a linear probe was placed perpendicular to the long axis of the clavicle, in the deltopectoral groove, with the patient in supine position, allowing visualization of the vein and subclavian artery in the short axis (Figures 1c, 1d).

After locating IVC and SCV, the minimum (IVCmin or SCVmin) and maximum diameter (IVCmax or SCVmax) of IVC and SCV at the end of inspiration and expiration were measured in M-mode during the same respiratory cycle while the patient was breathing quietly [1,11]. Three values of each IVC diameter and SCV diameter were taken in all the patients and the mean of these three measurements was considered. Using these mean values, the CI was calculated [11].

$CI = \frac{D_{max\,exp} - D_{min\,insp}}{D_{max\,exp}} \times 100\%$

where D_max exp_ = Maximum diameter during expiration; D_min insp_ = Minimum diameter during inspiration

For each USG image, the time taken between probe placement and venous measurements made was noted by a nurse present at the time of evaluation [1]. The anesthesiologist administering the drugs in the operating room (OR) was unaware of the preoperative IVC and SCV findings. In the OR, ASA standard monitors were attached and baseline parameters (heart rate (HR), SBP, diastolic blood pressure (DBP), MAP and oxygen saturation) were recorded. Patients were preoxygenated with 100% oxygen for 3-5 minutes prior to induction of anesthesia. Subsequently, IV midazolam (0.02 mg/kg) and fentanyl (2-3 µg/kg) were administered as part of the standard induction practice routinely followed at our institution. Anesthesia was induced with titrated doses of IV propofol (1.5-2.5 mg/kg), followed by vecuronium (0.1 mg/kg) to facilitate endotracheal intubation using an appropriately sized endotracheal tube. Anesthesia was maintained with a mixture of oxygen and nitrous oxide (50:50), isoflurane (minimum alveolar concentration of 1%), and intermittent IV boluses of vecuronium (0.015 mg/kg). Non-invasive BP (NIBP) was monitored at the baseline (before administration of propofol), every minute for the first five minutes following propofol administration, and subsequently every two minutes for the next 10 minutes. Any tracheal intubation requiring more than three attempts was excluded from further data analysis because of sympathetic stimulation [19]. The patients remained supine and preparation of the surgical area was allowed during this period.

**Figure 2:**
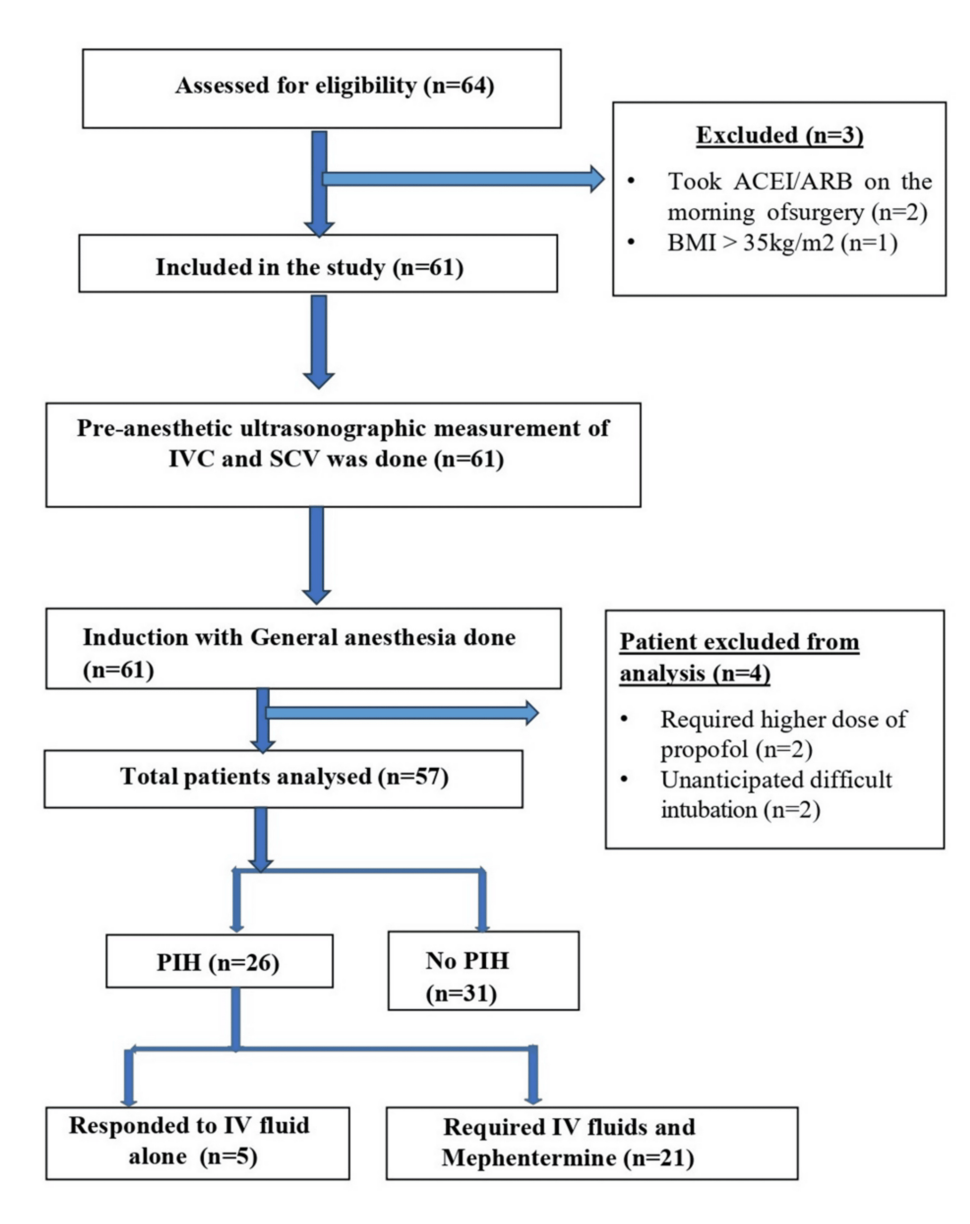
Strobe flow diagram of the study ARB: Angiotensin receptor blocker; ACEI: angiotensin-converting enzyme inhibitor; BMI: body mass index; IVC: inferior vena cava; SCV: subclavian vein; PIH: post-induction hypotension; IV: intravenous

PIH was defined as a drop in MAP < 65 mmHg or a drop of more than 30% from baseline [4,6]. Any episode of PIH was treated with a fluid bolus of 5 ml/kg. Persistent PIH, defined as lasting ≥ 2 minutes after fluid administration or MAP < 55 mmHg, was treated with a 6 mg IV bolus of mephentermine. Any episode of bradycardia (HR < 50 beats/minute) was treated with atropine 0.6 mg IV. The patient recruitment process is summarized in Figure 2.

Statistical analysis

The sample size was calculated using G*Power 3.1, based on parameters from a previous study [20]. Assuming a medium effect size (η² = 0.06), a correlation of 0.5 among repeated measures, three measurements per subject, non-sphericity correction of 0.5, 90% power, and α = 0.05, the required sample size was 57 [10].

The statistical analysis was conducted using IBM SPSS Statistics for Windows, Version 29 (Released 2023; IBM Corp., Armonk, New York, United States) and RStudio Team 2020 (RStudio: Integrated Development for R. RStudio, PBC, Boston, MA URL http://www.rstudio.com/). Categorical data were presented as frequencies (percentages) and continuous variables as mean (standard deviation) and were analyzed using the chi-square test or Fisher’s exact test and the independent Student’s t-test, respectively. Receiver-operating characteristic (ROC) curves, along with area under the curve (AUC) with 95% confidence interval, were used to evaluate the diagnostic accuracy of IVC and SCV CI in predicting PIH following GA. Various diagnostic indices, such as sensitivity, specificity, positive predictive value (PPV), negative predictive value (NPV), and misclassification rate, were calculated. The optimal cut-off value for each diagnostic variable was determined based on the Youden index (sensitivity + specificity - 1).

## Results

Sixty-four participants were assessed for eligibility in our study after obtaining written informed consent. Seven patients were excluded for various reasons as specified in the Strobe flow diagram (Figure 2).

Out of the 57 analyzed patients, 26 patients (45.61%) developed PIH following GA. Comparison of baseline characteristics between patients with or without PIH is shown in Table 1. The distribution of surgical procedures performed is summarized in Table 2. Laparoscopic cholecystectomy was the most common procedure (52.6%), followed by hysterectomy (12.3%) and other procedures accounted for 21.1% of cases.

**Table 1:** Comparison of patient’s demographic data with or without hypotension after induction of general anesthesia Hypotension group (n = 26); No hypotension group (n = 31). Values are presented as number (%) for categorical variables. A p-value < 0.05 was considered statistically significant. *Statistically significant.

Variables		Hypotension		p-value
		Yes (n=26)	No (n = 31)	
		n (%)	n (%)	
Gender	Female	17 (65.4)	22 (71.0)	0.652
	Male	9 (34.6)	9 (29.0)	
Age (years)	18-30	1 (3.8)	9 (29.0)	0.010*
	31-40	4 (15.4)	10 (32.3)	
	41-50	11 (42.3)	7 (22.6)	
	51-60	10 (38.5)	5 (16.1)	
Body mass index (BMI) (kg/m^2^) Asian classification	Normal	2 (7.7)	12 (38.7)	0.025*
	Overweight	11 (42.3)	8 (25.8)	
	Obese I	13 (50.0)	11 (35.5)	
American Society of Anesthesiologists (ASA) class	I	11 (42.3)	26 (83.9)	0.001*
	II	15 (57.7)	5 (16.1)	
Nil Per Os (NPO) hours	6-10	7 (26.9)	14 (45.2)	0.046*
	10-12	7 (26.9)	12 (38.7)	
	>12	12 (46.2)	5 (16.1)	
Hypothyroidism	yes	2 (7.69)	1 (3.32)	
Diabetes mellitus	yes	5 (19.23)	7 (22.58)	
History of hypertension	No	15 (57.7)	28 (90.3)	0.004*
	Yes	11 (42.3)	3 (9.7)	

**Table 2:** Type of surgery performed among the study population Values are presented as number (%). # Other individual procedures include right hemicolectomy, Heller’s myotomy with Dor fundoplication, cystopanendoscopy with internal urethrotomy, myomectomy, percutaneous nephrolithotomy, appendicectomy, Frey’s procedure, gastrojejunostomy, open pancreatectomy with splenectomy, laparoscopic cystectomy with cholecystectomy, and laparoscopic aspiration.

Surgery type	n (%)
Laparoscopic cholecystectomy	30 (52.6)
Total abdominal hysterectomy/laparoscopic hysterectomy	7 (12.3)
Hernia repair (mesh hernioplasty, subcutaneous onlay laparoscopic approach)	4 (7.0)
Radical nephrectomy/nephrectomy	3 (5.3)
Diagnostic laparoscopy	2 (3.5)
Other individual procedures#	11 (19.3)

Patients with PIH had a significantly lower IVC average minimum diameter and higher IVC-CI and SCV-CI (Table 3). However, no significant differences were observed in the IVC average maximum, SCV minimum and the maximum diameters between patients with or without PIH.

**Table 3:** Comparison of hemodynamic parameters and pre-anesthetic IVC and SCV findings with or without hypotension after induction of GA Values are presented as mean (standard deviation). A p-value < 0.05 was considered statistically significant. *Statistically significant. IVC: inferior vena cava; SCV: subclavian vein; CI: collapsibility index; GA: general anesthesia

Variables	Hypotension		p-value
	Yes (n=26)	No (n=31)	
	Mean (SD)	Mean (SD)	
Avg. min. diameter of IVC (cm)	1.108 (0.305)	1.424 (0.535)	0.008*
Avg. max. diameter of IVC (cm)	1.727 (0.483)	1.761 (0.573)	0.812
IVC-CI (%)	34.791 (11.968)	20.250 (7.806)	<0.001*
Avg. min. diameter of SCV (cm)	0.384 (0.158)	0.477 (0.192)	0.054
Avg. max diameter of SCV (cm)	0.688 (0.220)	0.624 (0.219)	0.137
SCV-CI (%)	43.532 (14.960)	23.590 (10.840)	<0.001*

**Figure 3:**
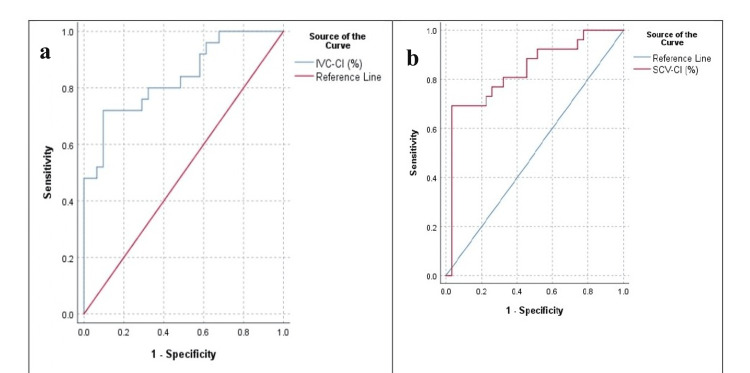
Receiver operating characteristic (ROC) curves showing the predictive ability of (a) inferior vena cava collapsibility index (IVC-CI) and (b) subclavian vein collapsibility index (SCV-CI) for detection of post-induction hypotension following induction of general anesthesia

The volume of IV fluids administered during the first 15 minutes after induction was significantly higher in patients with PIH compared to those without PIH (303.89 ± 95.134 ml vs. 255.74 ± 60.45 ml; p = 0.0243). The time taken to measure USG-guided SCV parameters was significantly less compared to that of IVC (111.58± 25.91 vs. 155.91 ± 35.77 seconds; p < 0.001). The AUC for IVC-CI was 0.836 (95% confidence interval: 0.730-0.957, p < 0.001) (Figure 3a) and for SCV-CI, it was 0.848 (95% confidence interval: 0.739-0.957, p < 0.001) (Figure 3b).

The optimal cut-off value of IVC-CI and SCV-CI for predicting PIH based on maximizing the Youden Index along with their sensitivity, specificity, PPV and NPV is mentioned in Table 4. The findings suggested that both IVC-CI and SCV-CI have comparable predictive capabilities for identifying patients at risk of developing PIH following GA.

**Table 4:** Comparison of receiver operating characteristic (ROC) parameters of IVC and SCV for predicting post-induction hypotension following induction of general anesthesia A p-value < 0.05 was considered statistically significant. *Statistically significant. AUC: area under the curve; IVC-CI: inferior vena cava collapsibility index; SCV-CI: subclavian vein collapsibility index; PPV: positive predictive value; NPV: negative predictive value

Variable	AUC (95% Confidence interval)	p-value (ROC)	Cut off index	Youden Index (%)	Sensitivity (%)	Specificity (%)	PPV (%)	NPV (%)	Misclassification on rate (%)
IVC-CI (%)	0.836 (0.730-0.957)	<0.001*	27.91	62.3	73.1	90.3	86.3	80	17.5
SCV-CI (%)	0.848 (0.739-0.957)	<0.001*	35.59	66.2	69.2	97%	95.8	75.8	16.9

**Figure 4:**
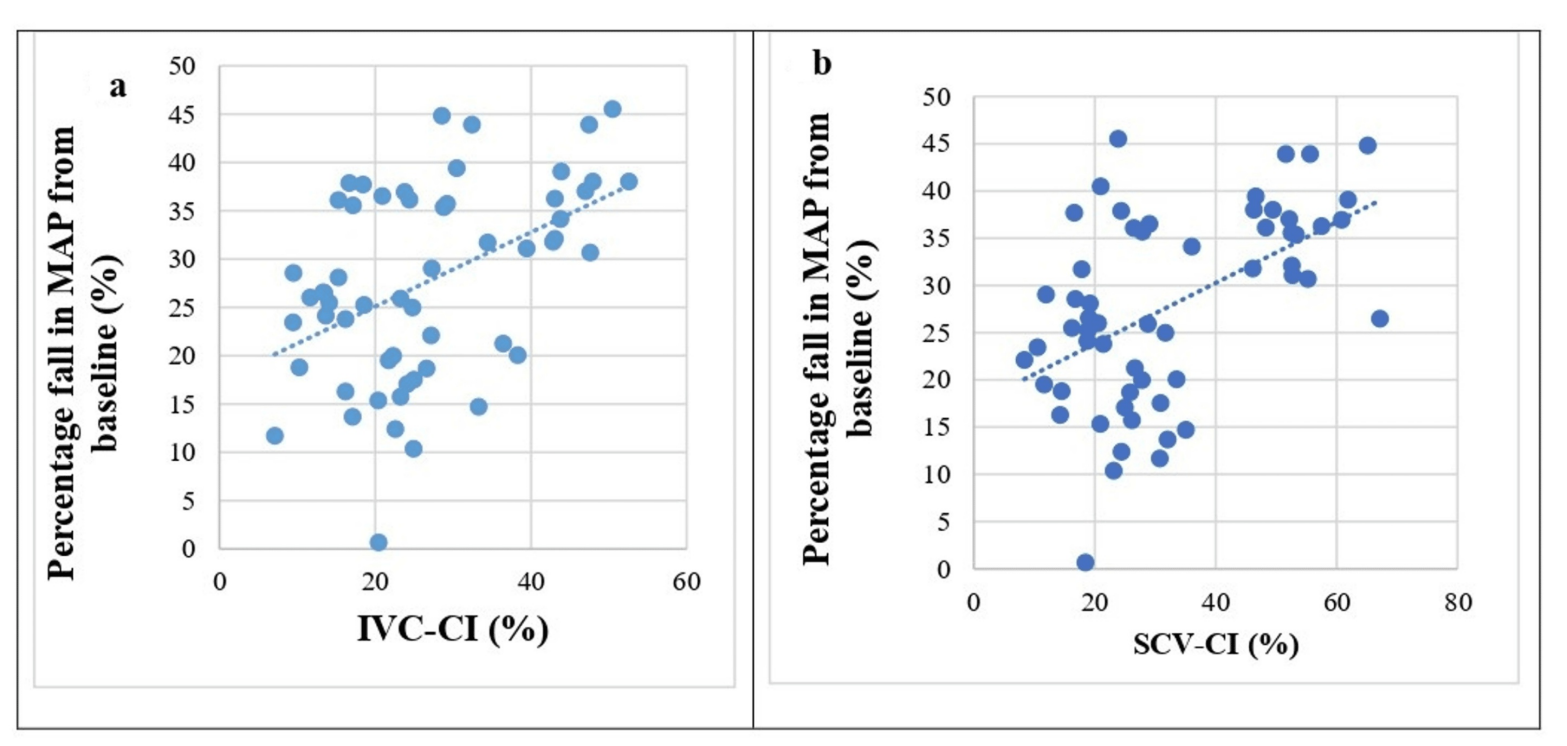
Scatter plots depicting relationship of (a) Inferior vena cava collapsibility index (IVC-CI) and (b) subclavian vein collapsibility index (SCV-CI) with percentage fall in mean arterial pressure (MAP) from baseline following induction of general anesthesia

The scatter plot in Figure 4 depicts the relationship between IVC-CI (Figure 4a) and SCV-CI (Figure 4b) with the percentage fall in MAP from baseline following induction of GA. Each point on the plot represents an individual data point, correlating the respective CI with the change in MAP.

**Figure 5:**
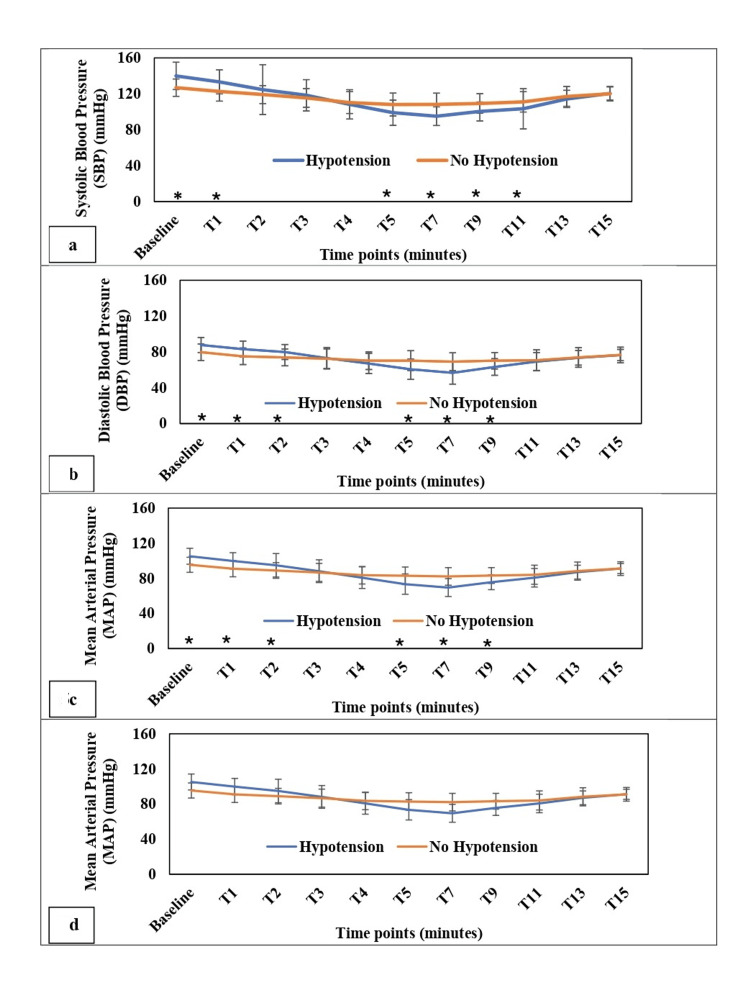
Trends in hemodynamic parameters over time in patients with and without post-induction hypotension Line diagram depicting changes in: (a) Systolic blood pressure (SBP), (b) diastolic blood pressure (DBP), (c) mean arterial pressure (MAP), and (d) heart rate (HR) measured at various time points after induction of general anesthesia (GA). Baseline represents measurements taken before induction of general anesthesia (GA). T1 and T2 represent one minute and two minutes after induction of GA, respectively, and subsequent time points (T3, T4, T5, T7, T9, T11, T13, and T15) represent corresponding minutes after induction. Values plotted are mean and standard deviation. *Statistically significant difference between groups (p < 0.05) at that particular time point.

The Pearson correlation coefficient prediction of PIH was statistically significant for IVC-CI (0.464) and SCV-CI (0.515). Figure 5 depicts SBP, DBP, MAP and HR readings at various time points among patients with or without PIH.

## Discussion

Our study findings demonstrate that the IVC-CI, SCV-CI, and average minimum diameter of IVC have significant predictive ability for identifying patients at risk of developing PIH following GA. The literature comparing IVC and SCV diameter and CI in predicting PIH is scarce. Rose et al. and Chaudhary et al. evaluated IVC and SCV parameters in different surgical populations such as gynecological, ear, nose, and throat, urological, spinal, general, and neurosurgical cases under GA [11,14]. Such heterogeneous surgical populations differ in patient positioning, stress, and physiological responses. In contrast, we studied exclusively in patients undergoing elective abdominopelvic surgeries, providing a more uniform cohort for analysis. Chen et al. compared them in elderly patients undergoing gastrointestinal surgeries, where age-related vascular changes, impaired baroreceptor reflexes, and comorbidities could alter the hemodynamic response to anesthesia [15]. They found that IVC-CI and SCV-CI were positively correlated with PIH. Kaptein et al. focused on critically ill patients with heart failure or kidney disease [12,13]. These populations are prone to extreme fluid shifts with unstable hemodynamics, volume overload, and altered vascular compliance due to the associated co-morbidities. Their aim was to assess volume status and guide fluid management in critical conditions, where volume overload or depletion requires precise management, whereas our study population represents a more controlled and homogenous group, minimizing confounding factors related to comorbidities and hemodynamic instability, and focused on predicting PIH [13].

In the present study, the average minimum diameter of IVC was statistically significant in predicting PIH, which concords with the findings of Rose et al. [11]. However, no significant correlations were found between maximum IVC diameter, maximum and minimum diameter of SCV with PIH following GA, which is in contrast to the results of Rose et al. [11]. Later, it was found that the SCVmax and SCVmin diameters of 0.69 cm and 0.70 cm during spontaneous and deep breathing, respectively, were statistically significant in predicting PIH [11]. Similar contradictory results were also seen in other studies where SCVmax and SCVmin diameter were found to significantly predict PIH [21,22]. The lack of correlation between the maximum IVC diameter or SCV diameters (both maximum and minimum) with PIH following GA in our study could be due to various factors. Firstly, the dynamic nature of these measurements could be affected by several patient-specific variables, such as venous system compliance, autonomic regulation, respiratory mechanics, associated co-morbidities, and overall hemodynamic status [22-24]. Also, the differences in the patient population and surgical procedures between studies might explain the observed discrepancies. Furthermore, the optimal cut-off index for predicting PIH was 27.91% and 35.59% for IVC-CI and SCV-CI, respectively. At these thresholds, the sensitivity and specificity were 73.1% and 90.3% for IVC-CI and 69.2% and 97% for SCV-CI, respectively, indicating acceptable diagnostic accuracy. Rose et al. found the cut-off value of 36% for SCV during deep breathing with sensitivity of 90% and specificity of 87% and the IVC cut-off value was 37% with sensitivity and specificity of 90% and 84%, respectively [11] and Chaudhary et al. found that during deep breathing, the cutoff value, sensitivity, and specificity for SCV-CI were ≥27%, 71%, and 51%, respectively, while for IVC-CI, they were ≥50%, 70%, and 56%, respectively [14]. However, the deep breathing maneuvers in their studies may have led to interindividual variability, as the depth and pattern of inspiration can differ among patients, influencing intrathoracic pressure and venous collapsibility. In contrast, the present study was conducted under spontaneous breathing, providing a more physiological and reproducible assessment of venous dynamics in elective abdominopelvic surgical patients. In the present study, SCV-CI showed higher AUC, specificity, PPV, and a lower misclassification rate compared to IVC-CI. This may be attributed to the relative anatomical stability of the SCV, which is less affected by variations in intra-abdominal pressure, bowel distension, or diaphragmatic excursion than the inferior vena cava. Additionally, SCV USG imaging is often technically easier and more reproducible, especially in patients undergoing abdominopelvic surgery. These findings indicate that SCV-CI could be considered a potential alternative to IVC-CI in similar elective surgical populations; however, further validation in larger cohorts is required to strengthen these observations.

Our analysis identified increasing age as a significant predictor of PIH. Age-related physiological changes like increased peripheral vascular resistance, altered adrenergic and vagal tone, predispose older patients to PIH [25,26]. In contrast, Rose et al. reported that there was no correlation between age (18-60 years) and PIH [11]. However, we found a statistically significant correlation within the same age group, with a higher mean age (46 years) among patients who developed PIH (p value = 0.010).

We did not find any association between gender and PIH as reported by previous studies [11,15,21]. Some studies suggest that women may have a higher incidence of PIH after GA, possibly due to physiological differences such as lower baseline MAP and hormonal influences [27,28]. However, we used Asian BMI classification for our North Indian population and observed that in the hypotensive group, 42.3% of patients were overweight (23-24.9 kg/m^2^) and 50% belonged to the obese I class (25-29.9 kg/m^2^). This is particularly relevant in North Indians, who have higher visceral adiposity and cardiometabolic risk at lower BMI compared to the Western population. The Asian BMI classification categorizes individuals as overweight and obese at lower BMI thresholds compared to the World Health Organization classification, thus reflecting different body composition and health risk profiles in Asian populations. This supports the use of Asian BMI criteria for more accurate identification of at-risk individuals in this population. Contrary to our findings, other studies did not find any effect of BMI on PIH [1]. These discrepancies may be explained by differences in ethnicity, body composition, lifestyle factors, and genetic predisposition across study populations.

Preoperative NPO hours and bowel preparation affect the intravascular volume status of the patient, which in turn increases the susceptibility of PIH after administration of anesthetic agents [1,11]. As mentioned in the ASA fasting guidelines, the patients were advised to be NPO for six hours for solid food (non-fatty food) and 2 hours for clear liquids [17]. However, our study observed that the patients were NPO for more than six hours, even for clear liquids, thus reflecting that with increasing fasting hours, the incidence of PIH has increased significantly.

Hypertensive patients were found to be more prone to developing PIH. Nabbi et al. showed that the patients who had taken ARBs or ACEIs on the day of surgery had decreased or aberrant physiological responses to common intraoperative vasopressors such as ephedrine, phenylephrine, and epinephrine, causing refractory hypotension [29]. However, we excluded such patients from the study. The patients who developed PIH received a higher mean dose of propofol (122.69 ± 13.43 mg) compared to those who did not develop hypotension (114.84 ± 18.95 mg); the difference was not statistically significant (p = 0.074). This suggests that the total propofol dose was not significantly associated with the occurrence of hypotension in our cohort. Therefore, pre-induction venous ultrasound parameters may provide additional value in identifying patients at risk beyond the effect of the induction drug dose alone. The study also revealed significant differences at specific time points (1, 5, 7, 9, and 11 minutes for SBP; 1, 2, 5, 7, and 9 minutes for DBP and MAP) after induction of GA. These time points indicate a critical window period where close monitoring and timely intervention may be required. It was also seen that patients with higher baseline BP were more susceptible to PIH. These findings align with the literature suggesting that the first few minutes post-induction are crucial for predicting and managing PIH [30]. The comparative analysis showed no significant difference in HR between the two groups. It was found that one out of the 57 patients analyzed had an episode of bradycardia after induction of GA, effectively treated with IV atropine 0.6mg. Patients who developed PIH required a significantly higher volume of IV fluids within the first 15 minutes post-induction compared to non-hypotensive patients (303.89 ml vs. 255.74 ml; p = 0.031). Despite fluid administration, 80.76% of hypotensive patients required mephentermine, suggesting that vasodilation and reduced vascular tone, rather than hypovolemia alone, primarily contributed to PIH.

In our study, the time taken to measure three USG values of IVC (155.91 ± 35.77 seconds) was 40-45 seconds more than that for SCV (111.58 ± 25.91 seconds) (p < 0.001), consistent with previous literature [10,11,14]. Chaudhary et al. reported that SCV measurements required less time than IVC measurements during both quiet and deep breathing (2.37 ± 0.71 min vs. 3.2 ± 0.9 min and 2.14 ± 0.63 min vs. 2.83 ± 0.94 min, respectively; p < 0.001) [14]. Similarly, Rose et al. observed that a single IVC measurement (48.44 seconds) required more time than an SCV measurement (40.37 seconds) [11]. Kent et al. observed a comparable finding in SICU patients, with median measurement times of 90 seconds for IVC and 47 seconds for SCV [10]. Although our study was performed in elective surgical cases, this time difference can be advantageous in acutely critical conditions where even a single minute delay can cause a significant effect on the patient’s outcome. However, its actual benefit in improving outcomes in critical care settings remains unknown.

The results of the present study suggest that pre-induction USG assessment of SVC and IVC CI can be used as a bedside risk-stratification tool for PIH. Identification of patients with high IVC-CI or SCV-CI may allow anesthesiologists to cautiously titrate anesthetic agents, to initiate early vasopressors, or to target fluid optimization. Although these indices do not directly predict arterial pressure, they provide useful surrogate information regarding intravascular volume status and venous capacitance, which are key contributors to PIH.

Strengths and limitations

Strengths of the study were that a single anesthesiologist measured all the USG parameters of both IVC and SCV preoperatively, and another anesthesiologist, unaware of the preoperative USG finding, induced the patient in the OR and measured the vital parameters, thus reducing variability and enhancing the reliability of the results. We applied Asian BMI classification, which better reflects body composition and cardiovascular risk profiles in the North Indian population, enhancing the external validity of our findings.

Limitations were that the bias in trial design could not be entirely eliminated, as it was an observational study. Second, we restricted enrollment to ASA I-II patients and excluded ASA ≥III patients and patients undergoing emergency surgeries. Hence, our results should be validated in critically ill populations. Third, the study sample size was relatively small, and thus, it may affect the generalizability of our findings to a larger patient population. Also, the small sample size and limited number of outcome events prevented the use of multivariate regression analysis. Fourth, measurements were performed by a single experienced anesthesiologist, which ensured procedural consistency; however, formal assessment of inter-observer variability was not undertaken. Therefore, the reproducibility of these measurements across different operators requires further evaluation. Although a standardized induction protocol was intentionally used to minimize variability and reduce confounding related to anesthetic agents and dosing strategies, this approach restricts the generalizability of the findings to other induction techniques. The predictive performance of IVC and SCV indices under alternative anesthetic strategies remains to be established. Finally, our study focused on comparing these parameters during normal breathing within the same respiratory cycle, whereas previous studies have demonstrated that SCV-CI and IVC-CI can predict PIH during both quiet and deep breathing. However, deep breathing might be difficult or uncomfortable in patients with a distended or tensed abdomen, critically ill, or those with compromised respiratory function. Furthermore, venous CIs should be viewed as supportive tools for risk prediction rather than alternatives for continuous BP or comprehensive hemodynamic monitoring.

## Conclusions

Pre-induction USG parameters IVC-CI, SCV-CI, and the average minimum diameter of the IVC demonstrated significant diagnostic accuracy for predicting PIH in the North Indian population undergoing elective abdominopelvic surgeries under a standardized induction protocol. In addition to venous indices, age, BMI, and known hypertensive patients were associated with increased risk of hemodynamic instability following induction. Identification of such patients may facilitate anticipatory management strategies, including pre-induction intravascular volume optimization, careful titration of induction agent, early vasopressor use, and close hemodynamic monitoring. The USG-guided SCV visualization required less time as compared to USG-guided visualization of IVC. SCV-CI may be a feasible alternative when IVC visualization is difficult or when rapid assessment is needed. However, its applicability in such scenarios requires validation in larger, multicenter studies with more diverse patient populations and under different anesthetic techniques.
